# General Surgery Residents Improve Efficiency but Not Hospital Length of Stay in Trauma Care

**DOI:** 10.7759/cureus.52499

**Published:** 2024-01-18

**Authors:** Ryan Bacchus, David Levy, Christine Hickey, Abenamar Arrillaga, Christopher Raio

**Affiliations:** 1 Emergency Medicine, Good Samaritan University Hospital, West Islip, USA; 2 Surgical Critical Care, Good Samaritan University Hospital, West Islip, USA

**Keywords:** teaching and training residents and medical students, ed length of stay, average length of hospital stay, surgery residents, trauma surgery

## Abstract

Background

Good Samaritan University Hospital (GSUH) has been preliminary approved to become a Level I Trauma Center. The American College of Surgeons (ACS) requires Level I Trauma Centers to have senior surgery residents on the trauma service. To fulfill this requirement, GSUH has established an affiliation with Stony Brook University Hospital, a tertiary care hospital with an Accreditation Council for Graduate Medical Education-approved five-year postgraduate training program in General Surgery, to have senior surgery residents from their training program rotate and provide care to trauma patients beginning in July of 2021. Numerous studies over the past few decades have shown conflicting results on patient outcomes with resident involvement. A majority of the studies published only evaluated residents who were native to the respective hospitals. Our study evaluated the impact of surgery residents visiting from an outside hospital on hospital length of stay (LOS) in admitted trauma patients. As increased hospital LOS is strongly associated with increased hospital-acquired complications, increased healthcare costs, and poor patient experience, we used this to evaluate the efficiency of our trauma team with the addition of visiting surgery residents.

Methodology

A retrospective study was conducted utilizing the hospital’s trauma registry. Patients were divided into two groups: the first two years before the addition of surgery residents from July 1st, 2019, to June 30th, 2021, and the second two years after the addition of surgery residents from July 1st, 2021, to June 30th, 2023. The primary outcome measured the hospital LOS between the two groups. Pearson’s chi-square test was used to analyze all categorical data, and a t-test was used to compare differences in means.

Results

From July 1st, 2019, to June 30th, 2023, a total of 7,081 patients were admitted to the trauma service: 3,411 in the group with no surgery residents, and 3,670 patients in the group with residents (p = 0.052). The primary outcome, hospital LOS, was not significantly affected by the addition of surgery residents to the trauma service. Hospital LOS before surgery residents was 4.40 days compared to with residents at 4.41 days (p = 0.944). Mortality was significantly decreased with resident involvement at 1.9% compared to no residents at 2.7% (p = 0.017). Interestingly, the Emergency Department LOS was significantly longer in the group with residents, 268.82 minutes vs. 232.19 minutes (p = 0.004). The average New Injury Severity Score was 9.02 in the group with no residents and 9.04 in the group with surgery residents (p = 0.927). The majority of traumas in both groups were blunt trauma 96.5% with no residents vs. 97.1% with residents (p = 0.192).

Conclusions

The addition of visiting surgery residents to the trauma team did not significantly increase hospital LOS. Ultimately, having visiting residents on the trauma service may enhance resident education without compromising hospital LOS. Training at different hospitals can allow residents to experience different patient populations and different hospital protocols, making them adaptable and more prepared to work in different hospital settings, whether academic or community. Hospitals without their own residency programs could potentially form affiliations with residency programs to meet the ACS requirements, which can bring more patients to their hospitals.

## Introduction

The American College of Surgeons (ACS) requires the presence of a surgery resident on the trauma service for an institution to be verified as a Level I Trauma Center. Numerous studies over the past few decades have shown conflicting results on patient outcomes with resident involvement. For example, Offner et al. in 2003 showed that surgery residents decreased length of stay (LOS) in the emergency department (ED) and hospital while not affecting mortality [1]. Another study by Taylor et al. in 2005 compared trauma outcomes in hospitals with emergency medicine (EM) residencies to those without and found that EM residents improved trauma patient outcomes [2].

Conversely, other studies have shown no change in outcomes of trauma patients where residents participate in care. For example, a study by Gorelik et al. in 2018 performed at a Level II Trauma Center found no change in complications or mortality in trauma patients six months after starting a surgery residency program [3]. A more recent study at a Level I Trauma Center in 2022 found no significant impact on LOS and mortality by EM residents. Patients did, however, have more diagnostic studies and therapeutic interventions [4]. In 2012, Matsushima et al. compared outcomes in trauma patients admitted during protected educational time where there was no resident involvement [5]. This study found no significant differences in mortality, complications, or LOS. However, this study did show that the length of ED-LOS in trauma patients increased without residents.

A majority of the studies published have one substantial commonality: the evaluated residents were native to their respective hospitals conducting the study. Our study aims to evaluate the impact of surgery residents visiting from an outside hospital on hospital LOS on admitted trauma patients. As increased hospital LOS is strongly associated with increased hospital-acquired complications, increased healthcare costs, and poor patient experience, we believe this will allow us to evaluate the efficiency of our trauma team with the addition of visiting surgery residents [6].

## Materials and methods

Setting and participants

Good Samaritan University Hospital has established an affiliation with Stony Brook University Hospital to have senior surgery residents rotate and provide care to trauma patients beginning in July 2021. Since then, the trauma service has consisted of the following: one in-house attending at all times throughout the day, two advanced practice providers (APPs), and one senior surgery resident. There are currently two surgery residents training at Good Samaritan University Hospital at a time: a PGY-4 and a PGY-5. Typically, one of these two residents spends the day in the operating room (OR) while the other takes care of the trauma patients. These surgery residents have numerous tasks to perform, which include, but are not limited to, responding to trauma activations, seeing consults, performing procedures, assisting in the OR, and formulating plans for the patients under their care. All decisions regarding medical management, admission, and discharge are ultimately made by the attending physician, pre- and post-inclusion of surgery residents.

Study design and data collection

A retrospective cohort study was conducted utilizing the hospital’s trauma registry. All reported data in the trauma registry is collected by administrators who identify patients via a review of the electronic medical record admission logs, trauma service report sheets, and morning reports. According to the National Trauma Data Standards (NTDS), 80% of all data is finalized within 60 days of discharge. Registry data is validated by NTDS submissions, which are abstracted monthly for elements that affect risk adjustment, benchmarking, and abbreviated injury scale coding using a data validation form. Automated registry software also provides automated checks for NTDS and the New York State Department of Health to identify structural or logistical errors.

Inclusion criteria

Utilizing the data from the registry, our study included pediatric and adult patients of all ages, transfers from outside facilities, trauma-related death on arrival, failed resuscitations, in-house deaths, and all admissions to the trauma service from July 1st, 2019, to June 30th, 2023. Inclusion criteria followed the guidelines set by the NTDS.

Statistical analysis

Data extracted from the trauma registry included, age, gender, ethnicity, hospital length of stay, ED-LOS, intensive care unit length of stay (ICU-LOS), trauma type, readmissions within 30 days, second readmission, trauma response time, comorbidities, and mortality. The New Injury Severity Scale (ISS) was also used as a medical score to assess trauma severity. It correlates with mortality, morbidity, and hospital LOS. A score of greater than 15 is indicative of severe trauma. Trauma activation level was also included from the trauma registry which is used to identify patients with potentially life-threatening injuries. Level 1 refers to a patient who meets a mechanism of injury criteria with unstable vital signs, while Level 2 activations refer to patients who meet a mechanism of injury criteria with stable vital signs.

Patients were divided into two groups: the first two years before the addition of surgery residents from July 1st, 2019, to June 30th, 2021, and the second two years after the addition of surgery residents from July 1st, 2021, to June 30th, 2023. The primary outcome measured the hospital LOS between these two groups as documented in the NTDS trauma registry. Secondary outcomes examined ED-LOS, ICU-LOS, readmissions within 30 days, second readmission, trauma response time, and mortality. Pearson’s chi-square test was used to analyze all categorical data, and a t-test was used to compare differences in means. We compared each variable from two years without residents to two years with residents on the trauma team. A p-value <0.05 was considered statistically significant.

Ethical considerations

This study was reviewed and deemed institutional review board (IRB)-exempt by Good Samaritan University Hospital GME IRB.

## Results

From July 1st, 2019, to June 30th, 2023, a total of 7,081 patients were admitted to the trauma service: 3,411 in the group with no surgery residents, and 3,670 patients in the group with residents (p = 0.052). Among demographic data involving the group with surgery residents, there were significantly more pediatric patients (p = 0.005) and Hispanic patients (p = 0.000). There were also fewer Caucasian patients totaling 63% (p = 0.002). There were also fewer patients with at least one comorbidity in the group with residents (60.6%) compared to the group with no residents (64.3%) (p < 0.001). There were no other statistically significant differences in demographics. Demographic data are presented found in Table 1.

**Table 1:** Demographic data. ^1^: non-Hispanic OR = odds ratio; CI = confidence interval

Variable	No residents (7/1/19 to 6/30/21)	Residents (7/1/21 to 6/30/23)	OR (95% CI)	P-value
Number of cases	3,411	3,670		
Age (years), mean (SD)	55.04 (27.1)	53.77 (27.9)		0.052
Age (year)				
0–14	307 (9.0%)	404 (11.0%)	1.241 (1.070–1.462)	0.005
15–65	1,692 (49.6%)	1,785 (48.6%)	0.962 (0.876–1.056)	0.416
More than 65	1,412 (41.4%)	1,481 (40.4%)	0.958 (0.871–1.053)	0.373
Gender				
Male	1,830 (53.6%)	1,954 (53.2%)	0.984 (0.896–1.080)	0.731
Female	1,581 (46.4%)	1,716 (46.8%)	1.017 (0.926–1.116)	0.731
Race/Ethnicity				
White^1^	2,269 (66.5%)	2,313 (63.0%)	0.857 (0.777–0.945)	0.002
Black^1^	375 (11.0%)	373 (10.2%)	0.916 (0.787–1.066)	0.256
Hispanic	552 (16.2%)	754 (20.5%)	1.339 (1.186–1.512)	0.000
Asian	40 (0.9%)	46 (1.3%)	1.431 (0.901–2.271)	0.127
Other	183 (5.4%)	183 (5.0%)	0.926 (0.750–1.143)	0.472

Hospital LOS before surgery residents was 4.40 days compared to with residents at 4.41 days (p = 0.944). ED-LOS was significantly longer in the group with residents, 268.82 minutes vs. 232.19 minutes (p = 0.004). For blunt traumas, ED-LOS was longer with residents at 273.07 minutes vs. 236.15 without residents (p = 0.005). Furthermore, patients admitted to the ICU had longer LOS in the ED at 201.69 minutes vs. 174.38 minutes (p = 0.002). Data for LOS are presented in Table 2.

**Table 2:** Length of stay.

Variable	No residents (7/1/19 to 6/30/21)	Residents (7/1/21 to 6/30/23)	P-value
All			
Number of cases	3,411	3,670	
Hospital length of stay, days, mean (SD)	4.40 (7.4)	4.41 (5.5)	0.944
Emergency department length of stay, minutes, mean (SD)	232.19 (208.5)	268.82 (735.0)	0.004
Intensive care unit length of stay, days, mean (SD)	6.59 (11.4)	6.00 (7.3)	0.270
Blunt trauma			
Number of cases	3,293	3,563	
Hospital length of stay, days, mean (SD)	4.35 (6.6)	4.39 (5.36)	0.784
Emergency department length of stay, minutes, mean (SD)	236.15 (210.0)	273.07 (745.2)	0.005
Intensive care unit length of stay, days, mean (SD)	6.28 (9.3)	5.90 (7.1)	0.418
Admitted to intensive care unit			
Number of cases	629	652	
Hospital length of stay, days, mean (SD)	9.76 (14.5)	9.44 (9.8)	0.647
Emergency department length of stay, minutes, mean (SD)	174.38 (139.2)	201.69 (168.5)	0.002
Intensive care unit length of stay, days, mean (SD)	6.59 (11.4)	6.01 (7.3)	0.278
Readmitted at least once			
Number of cases	38	76	
Hospital length of stay, days, mean (SD)	4.79 (4.6)	6.32 (7.4)	0.245
Emergency department length of stay, minutes, mean (SD)	253.00 (171.6)	215.09 (140.4)	0.210
Intensive care unit length of stay, days, mean (SD)	4.09 (4.3)	8.13 (9.4)	0.188

Secondary outcomes such as mortality and at least one readmission were also significantly different in the patients cared for in the group with residents. Mortality was lower in the group with residents at 1.9% compared to the group without residents at 2.7% (p = 0.017). There were also more readmissions in the group with residents at 2.1% compared to the group without residents at 1.1% (p = 0.001). ISS scores were nearly identical in both groups at 9.02 with no residents vs. 9.04 with residents (p = 0.927). The majority of traumas were blunt trauma in both groups at 96.5% in the group with no residents and 97.1% in the group with residents (p = 0.192). There were no other significant differences in secondary outcomes. Data for secondary outcomes are presented in Table 3.

**Table 3:** Secondary outcomes. OR = odds ratio; CI = confidence interval

Variable	No residents (7/1/19 to 6/30/21)	Residents (7/1/21 to 6/30/23)	OR (95% CI)	P-value
Trauma type				
Blunt	3,293 (96.5%)	3,563 (97.1%)	1.193 (0.915–1.557)	0.192
Penetrating	93 (2.7%)	85 (2.3%)	0.846 (0.628–1.139)	0.270
Burns	25 (0.7%)	18 (0.5%)	0.668 (0.364–1.226)	0.189
Transferred in	187 (5.5%)	196 (5.3%)	0.973 (0.792–1.195)	0.792
Trauma response time, minutes, mean (SD)	58.55 (116.1)	53.77 (126.3)		0.183
Activation level				
Level 1	247 (7.2%)	241 (6.6%)	0.900 (0.749–1.082)	0.263
Level 2	730 (21.4%)	765 (20.8%)	0.967 (0.863–1.084)	0.566
Admitted to intensive care unit	629 (18.4%)	652 (17.8%)	0.956 (0.847–1.078)	0.461
New Injury Severity Score, mean (SD)	9.02 (7.9)	9.04 (7.9)		0.927
Comorbidities				
At least one	2,193 (64.3%)	2,223 (60.6%)	0.853 (0.775–0.940)	0.001
Anticoagulant use	551 (16.2%)	554 (15.1%)	0.923 (0.812–1.049)	0.220
Mortality	93 (2.7%)	69 (1.9%)	0.684 (0.499–0.937)	0.017
Readmitted at least once	38 (1.1%)	76 (2.1%)	1.877 (1.268–2.779)	0.001
Total length of stay, days, mean (SD)	4.84 (6.3)	31.83 (231.5)		0.475
First readmission	38 (1.1%)	76 (2.1%)		0.001
Length of stay, days, mean (SD)	4.80 (6.3)	35.06 (244.7)		0.467
2^nd^ Readmission	3 (0.1%)	4 (0.1%)	1.239 (0.277-5.542)	0.778
Length of Stay, Days, Mean (SD)	5.33 (2.5)	8.75 (3.7)		0.229

## Discussion

In our retrospective cohort study, there were no statistically significant differences in hospital LOS with the inclusion of visiting surgical residents in admitted trauma patients. Our findings regarding hospital LOS are similar to other studies. For example, Matsushima et al. also found that there were no differences in hospital LOS in trauma patients with and without surgery resident involvement. McLaughlin et al. also found no statistically significant difference in hospital LOS when evaluating the impact of EM residents rotating on the trauma service [7]. Interestingly, ED-LOS was significantly longer in the group of patients with resident involvement in our study and in both studies mentioned above [5,7]. In our study, this can potentially be attributed to increased bedside teaching, more pediatric patients, and more patients requiring translator services as there were significantly more patients in these two groups. Secondary outcomes such as higher mortality in patients without resident involvement are likely because these patients were admitted during the height of the COVID-19 pandemic.

Despite the ACS requirements of having senior surgery residents on the trauma service for Level 1 verification, there does not seem to be a clear evidence-based benefit of this requirement in terms of improved outcomes. It can be deduced from our study that implementation of surgery residents does not lead to poor patient outcomes which is beneficial for resident education. Trauma patients require complex care starting from the initial encounter in the ED, to the OR, procedures, and floors. Although residents are explicitly involved in the care of these patients, most of the medical decision-making is made by trauma attendings in our department. The level of involvement of trauma residents may not have been enough to influence changes in patient-driven metrics. The Trauma Department has been run by trauma attendings and APPs for many years, which may have affected the amount of resident autonomy during their rotation.

There are several limitations of this study. First, the trauma registry is limited to data reported by the hospital. In the group of patients categorized by the addition of surgery residents, an assumption will have to be made that every patient had resident involvement. This is likely not the case as residents do have days off and/or have educational conferences to attend. Second, like other retrospective studies, we cannot establish cause and effect. Resident autonomy and skill level may vary depending on which PGY was assisting or due to attending discretion [8]. Additionally, each resident rotates on the trauma service for one to two months at a time, and therefore, certain factors can vary, such as their familiarity with hospital protocol, postgraduate year, experience, and level of competency. Lastly, the group categorized as before the addition of surgery residents was during the COVID-19 pandemic. The COVID-19 pandemic more than likely contributed to a significant increase in the mortality of patients.

## Conclusions

Ultimately, having visiting residents on the trauma service enhances resident education without compromising hospital LOS. Training at different hospitals can hopefully allow residents to experience different patient populations and different hospital protocols, making them adaptable and more prepared to work in different hospital settings, whether academic or community. Hospitals without their own residency programs could potentially form affiliations with residency programs to meet the ACS requirements, which can bring more patients to their hospitals.
